# Accelerating Anthropogenic Land Surface Change and the Status of Pleistocene Drumlins in New England

**DOI:** 10.1371/journal.pone.0046702

**Published:** 2012-10-09

**Authors:** Deborah W. Woodcock, John S. Rogan, Samuel D. Blanchard

**Affiliations:** 1 Marsh Institute, Clark University, Worcester, Massachusetts, United States of America; 2 Clark School of Geography, Clark University, Worcester, Massachusetts, United States of America; ETH, Switzerland

## Abstract

Drumlins are glacially derived landforms that are prominent in the landscape over much of southern New England. We carried out a comprehensive ground-based survey in a three-town study area in eastern Massachusetts with the goals of establishing the extent to drumlins have been altered and assessing the associated environmental consequences and probable driving factors. Results show that many drumlins have been significantly altered through levelling and truncation (creation of steep cut and fill slopes), with projects involving movement of 1−1.5×10^6^ m^3^ of earth materials not now uncommon. Stormwater and wetlands infractions were documented at all the larger excavation sites and resulted in enforcement actions and fines in many cases; the broader environmental consequences of the loss/alteration of these forested uplands are harder to establish. The excavations are significant in terms of materials cycling: the movement of earth materials, when considered regionally, greatly exceeds natural denudation processes and is also greater than during other periods of high anthropogenic denudation. Our findings suggest that the region’s glacial landscapes are at risk given current development patterns. The accelerating rate of land-surface change is undoubtedly also generalizable to other fast-developing regions of the United States. The landform alterations documented are part of a changing pattern of land use and vegetation cover since the Colonial era and are linked to shortages of land for development, current development and building practices, and lack of explicit rationales for preservation of the region’s geoheritage.

## Introduction

The drumlins of southern New England originated during the Wisconsinan (10,000–110,000 BP) and Illinoian (130,000–300,000 BP) glacial stages when they formed beneath massive ice sheets during the process of ablation [1], [2]. The landscape left after the ice retreated was hummocky with an abundance of bogs, swamps, and other wetland features and included extensive drumlin fields (Glacial Landsystem B of Colgan et al. [3]). The drumlins characteristic of this landscape are hills that are typically 15–30 m high and tear-drop shaped, tend to occur in groups or swarms, and can range in composition from till, stratified diamicton, and bedrock to fluvially derived materials [3]. Their formation appears to be a complex function of erosional, depositional, and deformational processes occurring when the ice sheets moved across permanently frozen ground near the ice margin [3].

Examples of drumlins being significantly altered in eastern Massachusetts motivated us to investigate this phenomenon and attempt to establish a context in which the significance of landform alterations could be assessed. One analysis we undertook showed that the excavations are extensive enough to be detected using remotely sensed data [4]. In conjunction with this analysis we also carried out a ground-based survey of drumlins in a three-town study area, the results of which are presented here. The goals of the survey were: 1) to establish the extent of modification to area drumlins, 2) to identify the probable driving factors, and 3) to evaluate the environmental significance of drumlin loss and alteration. It became evident over the course of the study that there were both proximal and more long-term environmental consequences that had to be considered. We document examples of the former, including wetland siltation, stormwater discharge into streams and water bodies, and slope failures stemming from the excavations.

Drumlin alterations are part of the extensive changes to the land cover and land surface of the earth brought about by human activities. The impacts of these changes on natural processes at a variety of scales are of some concern. Here we pay special attention to drumlin alteration as an example of human-mediated movement of earth materials, which has been estimated at three to four times rates earlier in geologic time [*5*]. Many of the activities contributing to movement of materials, as for example, filling and grading, are ubiquitous and difficult to quantify. Drumlins, as discrete features, allow for estimates of material moved in association with alterations, which can in turn serve as an indicator of the extent of land modification occurring more generally.

## Methods

The study area, encompassing two towns (Hudson and Stow) and one municipality with a city-type government (Marlborough), lies along a major interstate highway (I-495) that is part of the outer beltway system of the Boston metropolitan area. Our survey of the drumlins in this area relied on information compiled as part of the USGS Glacial Aquifer Systems Stratigraphy Project; USGS maps provide precise delimitations of sands/gravels versus till deposits and show drumlins as ellipsoid areas of thick till. We defined significant alterations as excavations involving cut slopes or fill slopes greater than 3 m in height or loss of >3 m in elevation. We carried out a field-based survey to establish the presence of significant modifications. Alterations (length, height, and degree of cut and fill slopes) were either measured by means of a hypsometer or determined through consultation of site plans in town records. We also determined the dates and types of building and development projects by accessing information in publically available on-line databases and town records.

Although wetlands protection and stormwater controls are mandated by state and federal law, there is no centralized database at the federal or state level with information about infractions or enforcement actions. To obtain this information, we consulted newspaper reports and records in offices of city or town Conservation Commissions, the Massachusetts Department of Environmental Protection, and US Environmental Protection Agency and interviewed town conservation officers and staff at state and federal regulatory agencies. Instances of enforcement actions and fines levied serve as indicators of the seriousness of violations of laws regulating stormwater and protecting wetlands. We also documented occurrence of slope failures where we could get this information; slope failures were sometimes but not always linked to stormwater/wetlands infractions.

**Figure 1:**
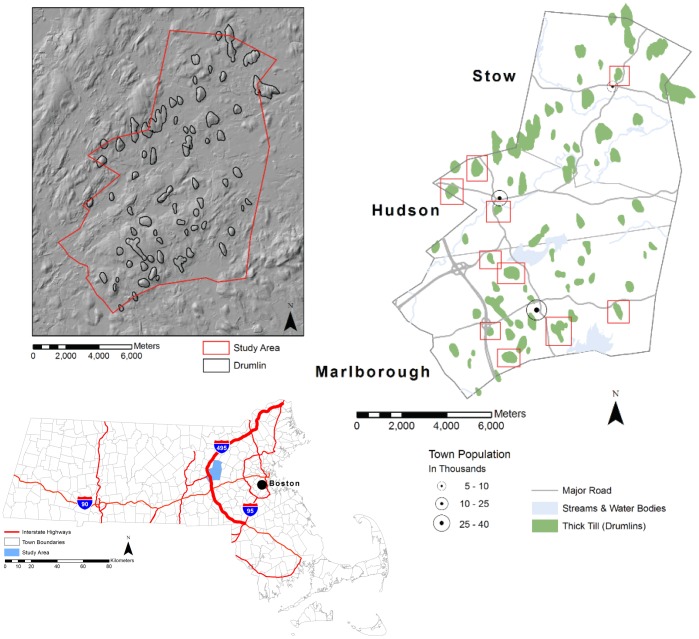
The three-town study area in eastern Massachusetts. Drumlins experiencing significant alterations are boxed in red (right). The area for the estimates of regional materials movement lies along that part of the Interstate 495 indicated by the thicker red line (lower left).

The volume of material removed from these geometrically fairly regular landforms was estimated from topographic maps based on the dimensions of the excavations. Estimates of regional movement of materials were made for a 25-town, 1308 km^2^ area along Interstate 495 (extending from Foxborough in the south to Amesbury at the northern terminus; see Figure 1) assuming a density of glacial till of 2.7 g cm^−3^ [6] and rates of development that seemed plausible based on trends seen in the study area and regionally. These estimates were then compared to denudation rates occurring naturally and during periods of increased sedimentation over the historical era.

No specific permits were required for the field surveys since the modifications were generally observable off site (from the road or other public land) and detailed information allowing estimates of cut slope characteristics could also be obtained from site plans filed with local municipalities. The field survey did not present a threat to endangered or protected species.

## Results

**Figure 2:**
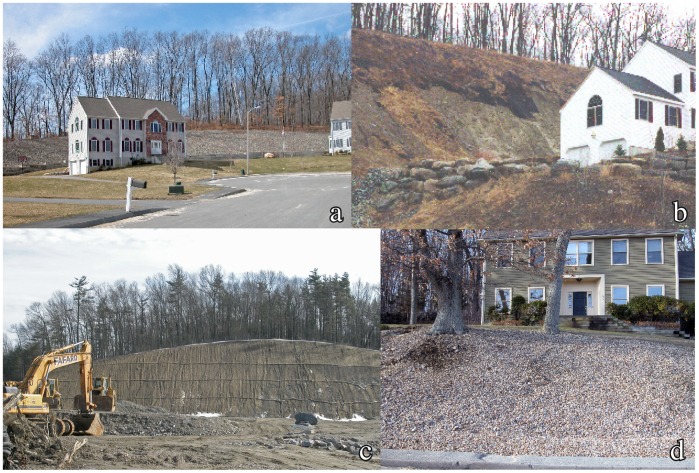
Drumlin modifications within the study area in eastern Massachusetts. A) An example of the large houses on relatively small lots associated with significant landform modifications. B) Slope failure on an excavated cut (Phillips Hill, Hudson). C) Large excavation of drumlin end (Indian Head Hill, Marlborough). D) Cut/fill slope on house lot (Jericho Hill, Marlborough).

The >60 drumlins in the study area include some hills with minor amounts of relief but also the majority of the area’s prominent landscape features. We found that 17% (10/60) of the landforms surveyed meet the criteria of experiencing significant modifications (Figure 1). The modifications include: 1) terracing of drumlin slopes and/or truncating of the features’ natural contours by cutting into the lower slopes, in some cases creating quite steep cut faces (eight examples), and 2) levelling of drumlins from the top down (two examples). The development projects associated with drumlin modification include four examples of single-family housing, two examples of multifamily housing (affordable and over-55 housing developments), one corporate office building, and two retail developments. With one exception, all date to the 1990s or after. There were also two examples of bedrock hills being significantly altered, one for single-family housing and one for multifamily housing. The excavations that are most significant in terms of material removed are three projects (Indian Head and Hager hills in Marlborough and Potash Hill in Hudson); the amount of material excavated in each of these projects is estimated at ∼1−1.5×10^6^ m^3^. Excavated cut slopes reach vertical heights of 23 m and slopes as high as 36° (Table 1), a considerable oversteepening relative to the natural contours of these features (which have slopes of 15–20° on their steepest sides). Some of these excavations are shown in Figure 2.

**Table 1:** Significant Alterations to Drumlins in the Central Massachusetts Study Area.

Drumlin &location	Development dates	Type of development	Type of alteration	Soil and tilltransported offsite (m^3^)	Cut/fill slope dimensions	Environmental problems	Regulatory actions
**Marlborough**							
Hill east of Framingham Rd 42°33′46.07 N 71°53′35.56 W	2002–04	single family housing	excavation of base of drumlin for house lots	–	cut slope 5–8 m high,18–21° with retainingwall at base	–	–
Shoestring Hill 42°33′34.27 N 71°55′50.65 W	1988–91	single family housing	excavation of upperslopes for house lots,road to top of drumlin	–	cut and fill slopes 3–6 m high, to 26°	failure to maintainerosion controls	enforcement action, two fines by town totaling $75
Jericho Hill northside 42°32′78.72 N 71°56′33.99 W	1988–92	single family housing	excavation of middle-upper drumlin slopesfor house lots, road	–	cut and fill slopes to 6–15 m high, 20–30°	siltation of wetland	enforcement action
Indian Head Hill 42°34′59.27 N 71°50′36.75 W	2007–08	big box store	excavation of one end ofdrumlin to create 6 acrelevel site, elevationloss of 20 m	1−1.5×10^5^ (est)	cut slope 23 m high, 220 m long, 25°	failure of erosion controls and detention system, flooding into nearby houses and businesses,silt discharge to pond andstream; slope failures	4 enforcement actions, 3 fines by town totaling $700; federal fine of $150,000 and order of $300,000 remediation project for stormwater violations at this and 12 other of developer’s construction sites
Hager Hill 42°36′81.49N 71°57′33.99 W	2008–09	over-55 housing	excavation of drumlinfrom top down to create13 ha leveled site, 13 mloss of elevation	1×10^5^ (est)	cut/fill slopes stabilized with retaining walls 4 m high, 90–95 m long	failure of erosion controls	enforcement actions associated with clearing of site?
West Hill 42°35′62.05 N 71°57′08.99 W	1997–98	single family housing	terracing of upperdrumlin slope forhousing lots	–	cut/fill slopes 9 m high, 100 m long, 25°	–	–
**Hudson**							
Phillips Hill 42°40′39.82 N 71°58′09 W	1998	single family housing	excavation of base of drumlin for house lots	–	cut slope 11–17 m high, 200 m long, 25–36°	failure of erosion controls; slope failure leading to threatened lawsuit, remediation of slope with stacked rock face.	federal fine of $3,350 for failure to develop Stormwater Pollution Protection Plan and inadequate erosion controls
Pope Hill 42°38′73.16 N 71°57′00.66 W	2008	apartment building	excavation of base of drumlin for level building site	–	cut slope stabilized with 6 m high retaining wall	sedimentation of catchbasins and river	enforcement action
Potash Hill 42°39′45.37 N 71°59′39.55 W	2007–08	shopping center with big box store, on-site sewage and stormwatertreatment	excavation of drumlinfrom top down to create∼12 ha level site; 18 mloss in elevation	1−1.5×10^5^ (est)	fill slope stabilized with 21 m high retaining wall	failure of erosion controls,overflow of catchments,siltation of brook and pond	4 enforcement actions; state and federal involvement
**Stow**							
Pilot Grove Hill 42°44′03.71 N 71°50′36.75 W	1989–90	affordable housing;on site septic andwell	excavation of one end of drumlin to create terraced site and for entryway;11 m loss inelevation	283 m^3^ soil, 4587 m^3^loam (Stow earthremoval permit)	cut slope 20 m high,30 m long, 34°; fill slope 9 m high, 150 mlong, 24°	discharge of stormwater/siltation of stream and water body associated with clear-cutting/removalof loam, breaching of containments,slope failure	8 enforcement actions, 13 fines by town totaling $2,600, and state fine of $21,625

The main environmental regulations relevant to the excavations are federal and state laws prohibiting stormwater from leaving building sites or being discharged to waterways, waterbodies, or wetlands. Information about environmental problems associated with alterations was not readily available owing to complexities in regulatory oversight, variations in record-keeping from town to town, and lack of reporting requirements and centralized records at the state level. It was possible, however, to document a high frequency of environmental problems in areas of drumlin excavation. Significant alterations were associated with problems and/or regulatory actions that included flooding of adjacent properties, stormwater infractions and violations of the Clean Water Act and Massachusetts Wetlands Protection Act, enforcement actions requiring extensive involvement of Conservation Commissions and officers, and fines at the local, state, and federal levels (Table 1). In some cases, these problems began when vegetation was stripped from the hills. In other cases, problems occurred later and were related to overflow of catchments and failure of erosion control measures with or without accompanying slope failure. There were several incidences of slope failures occurring after construction when they were particularly costly for owners and/or developers and in some cases resulted in lawsuits or threatened legal action. Although 1) many of the projects occurred during a time of rapid development when regulators may have had many matters demanding their attention and 2) regulatory responsibilities regarding stormwater are now greater at the town level, it still appears that large sites are very difficult to stabilize so that wetlands and watercourses are not adversely affected during excavation and before the areas are stabilized.

**Figure 3:**
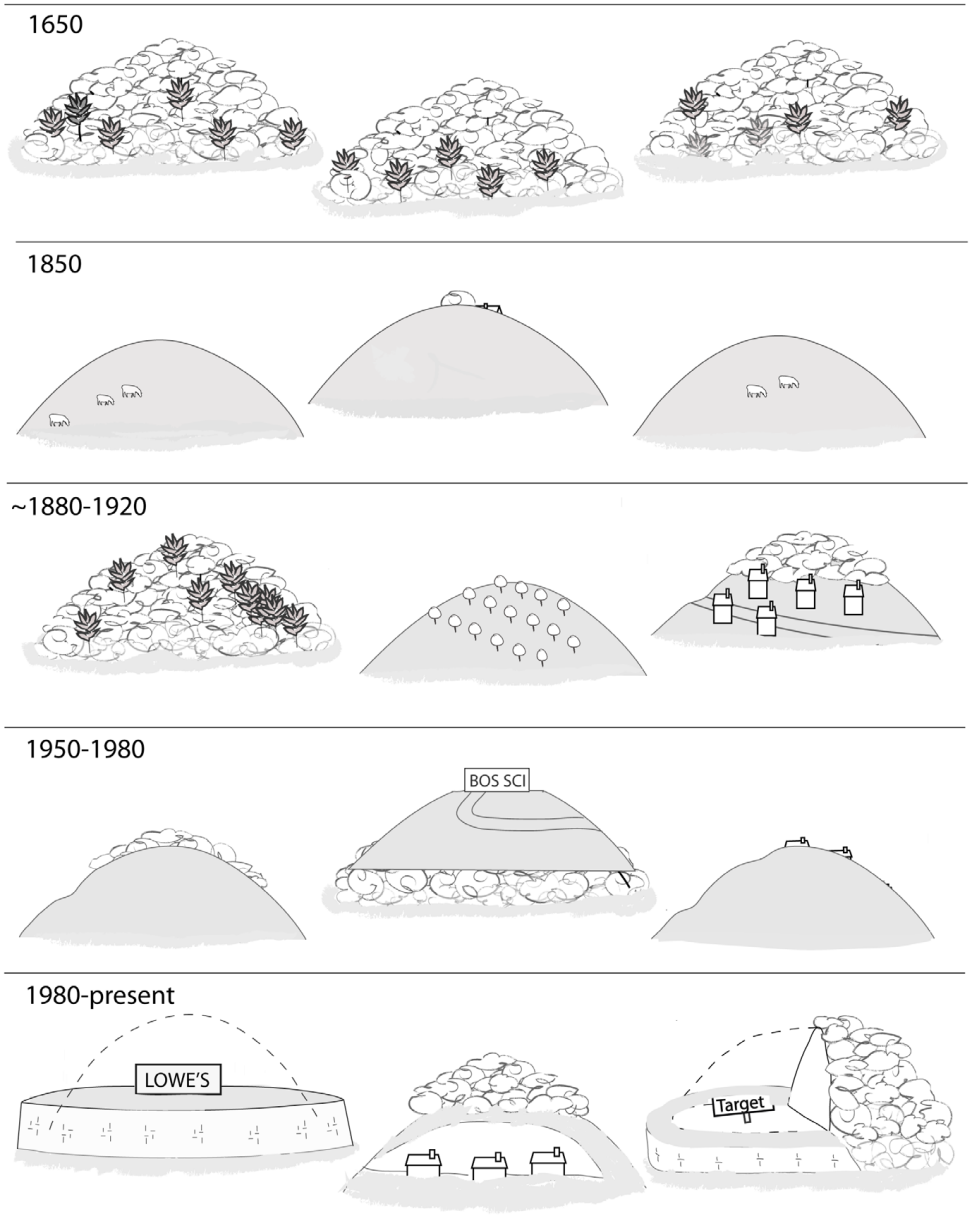
Diagram of the changes in eastern Massachusetts drumlins over time. See text for explanation.

The landform alterations documented in the study area are part of the most recent chapter of changing land use in New England and can be put into the historical framework established by studies such as [7] and [8]. The following periods can be recognized (Figure 3):

**1650.** Prior to European settlement, extensive forests covered a glacially derived landscape with abundant wetlands and surface waters.

**1850.** Clearing of forests for timber, agriculture, and sheep farming starting in the colonial era [7], [8] encompassed the region’s low hills, including many drumlins, by the mid-1800s. During this period, drumlins were often the location of potash operations producing fertilizer from wood ashes (thus the region’s many Potash Hills). In Boston, significant land-modification projects of the 1800s included filling in of Back Bay and harbor areas with material mined largely from nearby eskers [9] and grading, and in one case razing (Fort Hill), of a number of drumlins [10], [11].

**∼1880**–**1920.** Massachusetts’ forests have been regrowing since agricultural abandonment in the mid-1800s [7], [8]. By ∼1920, many area drumlins were becoming reforested. Orchards were also established on the tops and sides of drumlins [12], which are still considered ideal for this purpose [13]. In some areas of high population density, houses were built on lower drumlin flanks (Fairmont Hill in Marlborough).

**1950**–**1980.** Starting around 1950, housing developments were established on some drumlins where slopes permitted (typically involving some degree of grading and levelling). Exacavations for the Massachusetts Turnpike in the 1950s also involved shearing off the top of at least one drumlin (The Mountain in Framingham [14]) and significant excavations of others.

**1980-present.** The first examples of corporate offices and office parks being established on the tops of drumlins date to the High-Tech boom that started in the 1980s - notably Lucent Technologies and Boston Scientific on Addition Hill and 3-Com on Crane Hill (Marlborough) and Bose Corporation on The Mountain (Framingham). The significant alterations documented in this study - high cut and fill slopes and levelling to create flat plots – have been occurring since around 1990.

## Discussion

Drumlins are mainly composed of compact tills that have low permeability, stay seasonally wet, and have high runoff rates when vegetation cover is lost. The climate of New England, with its wet springs and high levels of precipitation through the year, adds to the difficulties in controlling runoff, especially at large construction sites. Weather and substrate are factors that combine with slope characteristics (angle, area, height of cut slope) to influence slope stability. The instances of slope problems documented here occurred on slopes ranging from 20° to 36° (Table 1). The risk of slope problems is undoubtedly also added to by the structure of many local drumlins, which consist of an upper, brownish till that is late-Wisconsinan in age underlain by an older till that is grayish and more compacted and dates from an earlier glacial era [6], [15]. One study of the hydrogeology of an eastern Massachusetts drumlin [6] found wide variation in hydraulic conductivity and a decrease in hydraulic conductivity with depth (associated with a change from vertical to radial groundwater flow above the brown:gray till contact), properties that must certainly affect slope processes.

Development projects in areas of “compact till” such as drumlins have been cited for difficulties in controlling erosion during site development, the tendency for wet-season flooding and dry season drought, pollution problems associated with low percolation rates, and increases in already high runoff rates and surface soil saturation when forest cover is lost (Sirgun Gadwa, wetlands consultant, Cheshire CT; http://preservelandinghill.blogspot. com/2008/09/water-woes-on-drumlins.html and personal communication). Planning oversight should, in this view, take into account the need for lower housing densities in areas of compact till and give preference to preservation as forested open space. The regulatory and permitting environment in Massachusetts, important in understanding both the types of building practices permitted and the spatial pattern of drumlin alterations, is summarized in Supporting Information S1.

In addition to proximal effects (siltation and stormwater problems), broader ecological consequences of drumlin alteration and loss (and loss of relief more generally) may include effects on continuity of forest cover and biological diversity and connectivity, especially where drumlins have persisted as isolated forested islands within developed areas. The gradational pattern produced is one in which materials are moved either highly locally (when excavated material is used for fill on-site) or over longer, but still rather localized, distances (when material is trucked off-site, usually to nearby locations within the same or neighboring towns). The environmental consequences of this type of material movement (for transport of soil pathogens, for instance) are largely unknown. Since drumlins have high runoff rates, conversion to hard surfaces is unlikely to have significant effects on groundwater recharge, although runoff patterns within watersheds could be changed. These projects do, however, contribute to the proliferation of storm water discharge structures (detention basins and ponds, storm water channels and swales, constructed wetlands, etc.) that are an increasingly visible part of the landscape.

Along with changes in land use, sedimentation rates in New England have varied considerably over the past 150–200 years. Naturally occurring rates of sedimentation in this region are low relative to values worldwide. Rates were 4−11×10^3 ^kg km^−2^ yr^−1 ^over much of the Holocene, and similarly low values typify forested areas with little disturbance [16], [17]. During the early agricultural period, loss of forest cover and cultivation increased sedimentation rates to 30–2200×10^3^ kg km^−2^ yr^−1^ [16]. The large land-moving projects associated with drumlin alteration are located along major arterials within the “sprawl frontier” of Massachusetts [18]. If every town in a 25-town area within the fast-developing area along Interstate-495 (Figure 1) experienced major land-modification projects at the rate seen in Hudson and Marlborough (a minimum of 1 major land-development project per 5-year period involving ∼1×10^6^ m^3^ of material), the total amount of material moved over a 5 year period would be ∼7×10^10^ kg. Averaged over the entire 25-town area, this figure corresponds to a rate of movement of materials of 10×10^6^ kg km^−2^ yr^−1^, a value that dwarfs naturally occurring processes (∼.01×10^6^ kg km^−2^ yr^−1^ [*16]*, [17]) and also exceeds that occurring during previous periods of anthropogenically accelerated sedimentation (.03−2×10^6^ kg km^−2^ yr^−1^ [*16*]) while not taking into account the more generalized land-moving activities that are also ongoing.

Studies of anthropogenic sediment and movement of materials generally deal with soil loss from cropland or cleared areas and subsequent deposition of materials in lower-lying areas and river systems. The type of earth-movement studied here does not involve a widespread significant sedimentary signature of this sort but is likewise a type of denudational process in which material is removed from uplands and deposited at lower elevation. Brown [19] used the term “technical denudation” to describe the “consumption” of earth materials required by industrialized society and made one of the few existing estimates of corresponding denudation rates on a global basis. His 1956 calculation of the rate of technical denudation that would be necessary to support a world population of 30 billion (3.3 mm per year or 3 m per millennium averaged over all the world’s land surface) is remarkable. Despite the high population estimate utilized, it demonstrates the magnitude of these anthropogenic processes, which at even a fraction of the rates estimated point clearly toward the unsustainability of these activities over the longer term. Brown’s [19] estimates were based on mining and extraction, not routine conversion of land for residential and commercial purposes, yet our estimates for movement of materials approximate Brown’s global estimate (3.8 vs. 3.3 mm yr^−1^). High rates of development-driven movement of materials are undoubtedly also occurring elsewhere in the United States, especially along major roadways, with environmental correlates and consequences that vary by region but in eastern Massachusetts are clearly having an effect on the region’s glacial landscapes.

The following factors may be important in facilitating or driving the increase in landform/land-surface alterations indicated: 1. Increased physical capacity to modify the land surface (availability of earth-moving equipment, etc.) 2. Shortages of land for development. High population densities and limited developable land given the state’s extensive wetlands may be driving land-modification just as they did in 1800s Boston [9]. Rates of land conversion/forest loss, however, exceed that of population growth [20], [21]. 3. The limited importance of drumlins in local hydrology/groundwater recharge. As areas where runoff predominates, drumlins are not classed with recharge areas that may receive special development oversight when land conversion is proposed. 4. The profitability of commercial developments and willingness of developers to assume the costs of land-moving and related requirements (stormwater control structures, even on-site sewage treatment in some cases) as well as make sizable direct payments to municipalities (increasingly a component of the development equation). 5. Variations in development and conservation goals from town to town, with some towns having protection of hills and high ground as an important part of conservation agendas while others have different conservation goals or are more development-minded. 6. Zoning. Many drumlins are zoned industrial or limited-industrial (designations that may relate to prior usage as orchards or persist from more industrialized times). In this case, big projects do not require public referenda as would be the case if rezoning were involved. 7. Current building and development practices. The large footprint of big-box stores; building of large houses on small lots; and subdivision design standards specifying maximum slopes for roadways (10% for example), but not excluding road development where slope angles are high, lead inevitably to significant excavations in areas of relief. 8. The lack of explicit protections for landscape features. Environmental Impact Statements require assessment of impacts on cultural, historical, and archeological resources, but not resources that are aesthetic, geological, or geomorphological. In some parts of the world, glacial landscapes enjoy formal protection from significant alteration and development, but these examples are mostly outside of the US. A notable exception is in Wisconsin, where glacial landscapes are the centerpieces of the Ice Age National Scientific Reserve and Ice Age Trail and counties such as Door County have zoning regulations aimed at preserving geologic features that forbid mining and limit development on drumlins [22].

It also appears possible that drumlins have gone from being areas exempted from development, for reasons including water availability/difficulty of pumping water to higher elevations and physical limitations on building, to being targeted for development as non-wetland areas in an environment where developable land is limited and large commercial developments are highly profitable. The prediction can thus be advanced that significant landform modifications of the type documented here will continue, especially near interstates and interstate exits and along major arterials and in areas where preservation of these features is not given priority.

### Conclusions

An important issue, and one that cannot be addressed here in any complete fashion, is the valuation of drumlins as landscape elements and whether they should be considered part of the region’s historical, aesthetic, and geological heritage. Our findings that drumlins are being altered in significant numbers raises the very real possibility that the glacial landscapes of New England are at risk. Additionally, these landform alterations are part of a broad pattern of significantly accelerated materials cycling with both short- and long-term environmental consequences that are incompletely known or appreciated. For these reasons, the phenomenon of landform/land-surface alteration requires additional review.

## Supporting Information

Supporting Information S1 Regulatory considerations.
(DOCX)

## Acknowledgments

The first author appreciates assistance from Paul Bierman, Nathaniel Brown, Patrick Colgan, Raymond Coveney, Richard Cadwgan, Leslie Demichelle, Allison Field-Juma, Sigrun Gadwa, Dale Hattis, Gary Hollands, Ali Hosseini, Jeffrey Kopf, Steve Lambert, Syed Hasan, Peter LeTourneau, Larry Lewis, Stephen McCauley, Don Parsley, Priscilla Ryder, James Skehan, and town and city staff in Stow, Marlborough, and Hudson.
