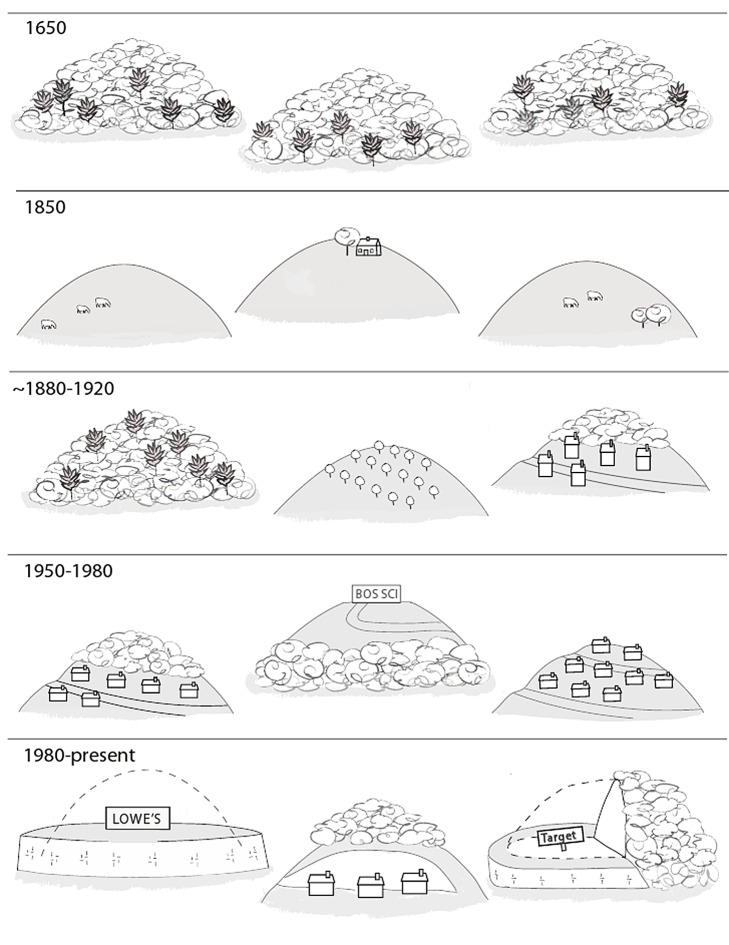# Correction: Accelerating Anthropogenic Land Surface Change and the Status of Pleistocene Drumlins in New England

**DOI:** 10.1371/annotation/e243021f-2ad9-4b2c-aa2b-2bbf5c056443

**Published:** 2012-11-06

**Authors:** Deborah W. Woodcock, John S. Rogan, Samuel D. Blanchard

There was an error in Figure 3. The correct figure can be viewed here: 

**Figure:**