# Supplementary material for: Accelerating Anthropogenic Land Surface Change and the Status of Pleistocene Drumlins in New England
Source: PLoS One. 2012 Oct 9;7(10):e46702. doi: 10.1371/journal.pone.0046702 (PMC3467288; doi:10.1371/journal.pone.0046702)
Supplement: Supporting Information S1 — Regulatory considerations. (DOCX) [file pone.0046702.s001.docx]

Supplementary Information S1.

Regulatory Considerations. Conservation Commissions in the Massachusetts’ 301 towns and 50 cities are responsible for enforcing the Massachusetts Wetlands Protection Act and issue an Order of Conditions and have enforcement authority for projects where wetlands could be adversely affected. Stormwater is under the more direct jurisdiction of the federal government (US EPA) through stormwater management plans reviewed in conjunction with Construction General Permits; larger municipalities (urbanized areas) also have review and enforcement authority for stormwater and related problems. Environmental Impact Statements, when required by the state because of a project’s size or potential adverse environmental effects, include an assessment of impacts on resources that are historic, archaeological, or cultural in nature. Slope-related problems are a matter for environmental oversight mainly to the extent that wetlands or stormwater discharge is affected. The Massachusetts Building Codes, as modified in 2009, permit cut and fill slopes greater than 27° (50% slope) only with a soil inventory report accepted by a local building official. Massachusetts cities and towns oversee and regulate building practices and make most development-related decisions and may or may not have guidelines beyond those set at the state level.

Town Conservation Commissions report to the Massachusetts Department of Environmental Protection. There is no reporting requirement for enforcement actions, although many municipalities do send this information to the DEP. State and federal agencies do not maintain databases of enforcement actions and this information is not readily available in any aggregate form. Not all towns (e.g., Hudson) are able to levy fines in cases of environmental infractions.
